# Random Access Performance of Distributed Sensors Attacked by Unknown Jammers

**DOI:** 10.3390/s17112667

**Published:** 2017-11-18

**Authors:** Dae-Kyo Jeong, Jung-Hwa Wui, Dongwoo Kim

**Affiliations:** 1 Department of Electronics and Communication Engineering, Hanyang University, Ansan 15588, Korea; dkjeong@wnl.hanyang.ac.kr; 2 Research Institute of Engineering and Technology, Hanyang University, Ansan 15588, Korea; jhwui@hanyang.ac.kr; 3 Division of Electrical Engineering, Hanyang University, Ansan 15588, Korea

**Keywords:** wireless sensor networks, random access channel (RACH), power jamming, code jamming

## Abstract

In this paper, we model and investigate the random access (RA) performance of sensor nodes (SN) in a wireless sensor network (WSN). In the WSN, a central head sensor (HS) collects the information from distributed SNs, and jammers disturb the information transmission primarily by generating interference. In this paper, two jamming attacks are considered: power and code jamming. Power jammers (if they are friendly jammers) generate noises and, as a result, degrade the quality of the signal from SNs. Power jamming is equally harmful to all the SNs that are accessing HS and simply induces denial of service (DoS) without any need to hack HS or SNs. On the other hand, code jammers mimic legitimate SNs by sending fake signals and thus need to know certain system parameters that are used by the legitimate SNs. As a result of code jamming, HS falsely allocates radio resources to SNs. The code jamming hence increases the failure probability in sending the information messages, as well as misleads the usage of radio resources. In this paper, we present the probabilities of successful preamble transmission with power ramping according to the jammer types and provide the resulting throughput and delay of information transmission by SNs, respectively. The effect of two jamming attacks on the RA performances is compared with numerical investigation. The results show that, compared to RA without jammers, power and code jamming degrade the throughput by up to 30.3% and 40.5%, respectively, while the delay performance by up to 40.1% and 65.6%, respectively.

## 1. Introduction

With the development of a wide range of intelligent and tiny wireless sensing devices, wireless sensor networks (WSNs) are being used for many applications in the fields of medical, industry, defense, smart homes, etc. In addition to these applications, WSNs currently drive Internet of Things (IoT) and machine-to-machine communications. WSNs consist of numerous sensor nodes (SNs) that are usually interconnected through radio frequency (RF) communication links.

Since the signal quality on a wireless medium such as RF easily degrades by unwanted interference, WSNs are vulnerable to malfunctioning SNs. In WSNs, attackers can create malicious SNs that can disrupt desired wireless transmission by generating interference and noise [1,2], which is so-called jamming [3]. Jamming attacks severely degrade the performance of WSNs, to a large extent, in energy consumption, throughput and delay.

Jamming attacks may be viewed as a special case of denial of service (DoS) attacks, which prevent or inhibit the normal use or management of communications through flooding a network with useless information using RF signals [4]. In [5], various techniques for detecting the presence of jamming attacks in WSNs are explored. In [6], jamming attacks and network defense policies are modeled together in order to capture the effect of knowledge available at each side. For LTE systems, jamming is also regarded as a major attack that causes significant DoS [7,8]. While most works have focused on the physical-layer aspects of jamming attacks, reference [9] evaluates the resilience of WSNs to jamming attacks in the aspect of the routing protocol.

On the other hand, jamming is also used to prevent eavesdroppers (Eves) from snooping legitimate communications, which is often called friendly jamming [10,11,12] or cooperative jamming [13,14]. The works in [10,12] investigate system-level issues of introducing friendly jammers. However, most works regarding cooperative jamming are in the direction of recently developing physical-layer security. In [13], multi-antenna jammers use null-space artificial noise (AN) that increases interference of Eves, but is avoided by the legitimate user, for which the right knowledge of the channel between the jammer and the Eves is essential. In [14], the cooperative jammers harvest energy transmitted by a source and use it to generate AN to jam the Eves in cognitive IoT networks, in which an auction framework that formulates the jammer selection and the power allocation together is also provided.

In WSNs, the messages are usually short and transmitted through random access (RA) procedures. However, to the best of our knowledge, there is a lack of detailed analysis in RA performance when attacked (or influenced) by jamming. In this paper, we model and investigate RA performance that is degraded by unknown jamming attacks. We assume that a central SN (referred to as the head sensor (HS) in this paper) collects information from distributed SNs through the RA channel (RACH). In order to avoid heavy collisions and contentions among crowded SNs, we assume a collision avoidance mechanism in which SNs transmit short preamble signals first and then send information messages only if they receive an indicator of acquisition (AI) of the preamble from HS. We assume a WSN that adopts direct sequence spread spectrum modulation like in ZigBee [15], IEEE 802.15.4 [16] and UMTS WCDMA [17] applications. Furthermore, two jamming attacks are considered: power and code jamming. In power jamming, attackers (if they are friendly jammers) generate noises and, as a result, degrade the quality of the signal from SNs. Power jamming is equally harmful to all the SNs that are accessing HS. To overcome the prevailing interference, so-called power-ramping by which SNs gradually increase transmitting power in preamble transmission is also modeled in this paper. Power jamming simply induces DoS without any need to hack HS and SNs.

On the other hand, code jammers need to know the boundary of access slots and the access code used to send preambles or the messages since they generate and send fake signals with the same spreading code being used by the legitimate SNs. The access slot can be known if the code jammers successfully look at the synchronization channel from HS and the access code is normally open in common air protocol specifications. Code jamming hence gives different effects compared with power jamming. At the stage of preamble transmission, code jamming reinforces the power level of SNs that use the same access code, but reduces, similarly to power jamming, the signal quality of SNs choosing different codes. As a result of code jamming, some SNs can receive AI more easily and have more chances to transmit the message, which is again jammed by the code jammers using the same code. Code jamming looks like RF spoofing where the jammers transmit a fake signal that masquerades as an actual signal [18]. The code jamming hence increases the failure probability in sending the information messages and intends to mislead the adequate allocation of radio resources.

In this paper, we also present the probability of successful preamble transmission with power ramping according to the jamming types and the resulting throughput and delay of information transmission of SNs, respectively. With numerical investigation, the effect of the two jamming attacks on RA performance of SNs is compared in terms of throughput, as well as delay. The results show that, compared to RA without jammers, power and code jamming degrade the throughput by up to 30.3% and 40.5%, respectively, while the delay performance by up to 40.1% and 65.6%, respectively.

The remainder of the paper is organized as follows. The system model, RA procedure, a power capture model according to jamming types and power ramping for preamble transmission are presented in Section 2. In Section 3, we present the success probability of preamble transmission, the throughput and the access delay. We compare RA performances of SNs attacked by power and code jamming with numerical investigation in Section 4. Finally, conclusions are presented in Section 5. The main notations used in this paper are listed in Table A1 in Appendix C.

## 2. System Model

### 2.1. System Description and RA Procedure

**Figure 1:**
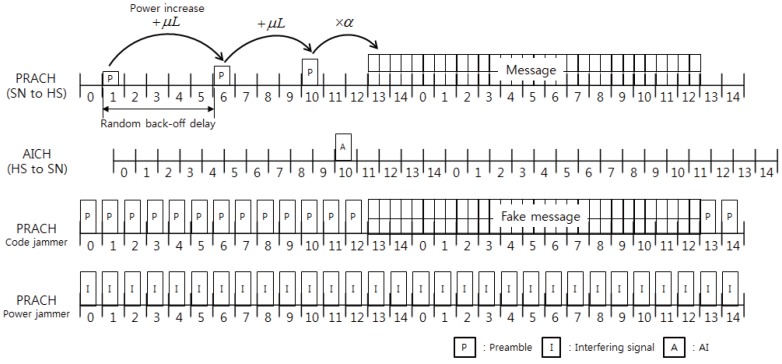
Mechanism of the RACH procedure.

We consider a cluster-tree-based WSN that consists of multiple noncooperative SNs and an HS. SNs measure certain (local, ambient) information and send it to the HS through an RF channel called a physical RACH (PRACH is a well-known terminology used in 3GPP standards. In this paper, however, PRACH does not imply a specific one used in current standards, but is a generic term that just indicates some physical-layer definitions for RACH described in the following). Every node is assumed to be equipped with a single omnidirectional antenna. We assume that a frame of PRACH is 20 ms long, which consists of 15 access slots, as in Figure 1. When an SN has information to send, a frame-long message (referred to as MSG) is assumed to encapsulate the information wholly and to be sent to the HS using PRACH. To send MSG successfully, the SN should completes three steps: transmitting a preamble on PRACH, receiving the AI of the preamble from the HS and sending MSG, which is referred to as a transmission cycle (TC) in this paper.

#### 2.1.1. RA Procedure

For a TC, SN starts with preamble transmission (PT). In PT, SN randomly selects an access code in a set of available orthogonal signature codes and an access slot among available slots. HS usually broadcasts a list of available codes and slots for SNs’ information. We assume that the preamble is 256 repetitions of the selected orthogonal spreading code of 16 chips long. It is noted that no user-specific information is carried by the preamble.

If HS receives the preamble successfully, it sends AI on the downlink (from HS to SN) AI channel (AICH in Figure 1). Unless the SN receives the AI adequately, it retransmits the same preamble again at a higher power level after a random back-off delay. Preamble retransmission will continue up to an allowable maximum number or until the preamble request is successfully received [17,19]. Parameter values used in PT (e.g., the transmission power range, the power control parameters and the maximum number of preamble retransmissions) are received through the higher layer control messages from HS.

If PT is successful and AI is correctly received by SN, MSG transmission (MT) will start using the same (selected) signature code used for PT and the same power level used in the latest PT. MSG usually delivers the information data, as well as the control data, which include pilot bits and/or the frame information. A TC is completed after successful PT and MT.

#### 2.1.2. Jamming Types

In power jamming, the jammer is assumed to send arbitrary signals or just sine-waves with full transmitting power at each slot. It causes the same level of interference to every SN in the system. On the other hand, in code jamming, the jammer is assumed to send a preamble using a randomly or specifically selected access code with the full power. It targets influencing the SNs using the same access code, but also works as interference for the other SNs. Since the code jammer works like a legitimate SN, it can hear AI from HS if PT is successful, and then, it sends a fake MSG using the same code, which further distorts the utilization of radio resources in the WSN.

#### 2.1.3. Traffic and RF Channel Model

The arrival of composite PTs (including initial transmissions and retransmissions) is modeled by a Poisson process with rate *G*, which is also known as the offered traffic. Let *N* be the number of (legitimate) SNs contending for the same access slot. The distribution of *N* is therefore:

(1) $$\begin{array}{c} \mathrm{Pr}\left\{N=n\right\}=\frac{G^{n}e^{-G}}{n!},\;n=0,1,2,\cdots . \end{array}$$

Let us denote a representative SN by SN-A (i.e., anonymous SN) for the purpose of presentation. Let *s* and *K* be the number of available access codes and the number of SNs selecting the same code with SN-A for accessing the same slot, respectively. Given *s* and N=n, the probability that SN-A collides with other K−1 SNs that choose the same code is given by:

(2) Pr{K=k|s,N=n}=n−1k−11sk−1s−1sn−k,1≤k≤n.

We assume that PRACH suffers from frequency-selective multipath Rayleigh slow-fading. Assuming that the shadowing and attenuation effects can be compensated by the open-loop power control used in UMTS RACH [19], the envelope of the received signal in one path is therefore a Rayleigh random variable. Let us assume that a perfect RAKE receiver with *L* fingers is used at HS. Additionally, we also assume that μ is the average received power from each path, and the received power of each finger $P^{\left(l\right)}$ has an exponential distribution, i.e., $f_{P^{\left(l\right)}}\left(x\right)=e^{-x/\mu }/\mu \;\left(x>0\right)$. Then, the signal powers distributed in *L* independent paths can be aggregated together so that the total received power $P=\sum_{l=1}^{L}P^{\left(l\right)}$ has a gamma distribution, i.e.,

(3) $$\begin{array}{c} f_{P}\left(x\right)=\frac{x^{L-1}e^{-x/\mu }}{\left(L-1\right)!\mu^{L}},\;x>0. \end{array}$$

It is noted that if we assume a narrow-band transmission like in narrow-band IoT, then PRACH can be assumed to suffer from a frequency-non-selective Rayleigh channel. In this case, L=1 in (3) builds the probability distribution.

### 2.2. Power Capture Model for Preamble Transmission

Let us consider a typical access slot where there are *s* access codes available and *k* SNs (including SN-A) select the same code. Let $n_{p}$ (previously *n*) denote the number of SNs that are in simultaneous PT, $n_{J}$ be the total number of jammers and $n_{m}$ be the number of SNs that are transmitting MSG in this slot. If code jamming is considered, we assume that there are one code jammer and $n_{J}-1$ power jammers. Let $P_{i}^{\left(p\right)}$, $P_{j}^{\left(m\right)}$ and $P_{J}$ be the total received power from SN-*i* transmitting a preamble, from SN-*j* transmitting MSG and from a hostile jammer, respectively. Since the preamble contains only the repetitions of a selected code, assuming a perfect RAKE receiver, HS can aggregate the power of PTs from those *k* code-collided SNs (including SN-A) and the jammer (if it uses code jamming) [20,21]. Let $\beta_{p}$ be the minimum signal-to-interference-plus-noise ratio (SINR) required to decode a preamble successfully. Then, the condition for correct reception of the preamble sent by SN-*A* (or SNs using the same code) when the attack is power jamming is:

(4) $$\begin{array}{c} \frac{P_{A}^{\left(p\right)}+\sum_{i=2}^{k}P_{i}^{\left(p\right)}}{\sum_{i=k+1}^{n_{p}}P_{i}^{\left(p\right)}+\sum_{j=1}^{n_{m}}P_{j}^{\left(m\right)}+n_{J}P_{J}+\eta }\geq \beta_{p}, \end{array}$$

where η represents interfering power from other sources (for example, from a neighbor WSN using an adjacent RF channel) plus background noise.

If the attack includes code jamming, the above condition becomes:

(5) $$\begin{array}{c} \frac{P_{A}^{\left(p\right)}+\sum_{i=2}^{k}P_{i}^{\left(p\right)}+P_{J}}{\sum_{i=k+1}^{n_{p}}P_{i}^{\left(p\right)}+\sum_{j=1}^{n_{m}}P_{j}^{\left(m\right)}+\left(n_{J}-1\right)P_{J}+\eta }\geq \beta_{p}, \end{array}$$

where $P_{J}$ in the numerator is the received power from the code jammer transmitting a fake preamble using the same code and $\left(n_{J}-1\right)P_{J}$ is the sum of powers from power jammers and/or the other code jammers that are using different access codes.

Let *q* denote the probability of successful reception of AI by an SN, which is assumed independent of all other uplink probabilistic matters since AI is received through the downlink. Additionally, let W be the number of SNs that successfully receive AI among K−1 SNs except SN-A. Then:

(6) Pr{W=w|K=k}=k−1wqw(1−q)k−1−w,0≤w≤k−1.

### 2.3. Power Capture Model for Message Transmission

When an SN receives AI successfully, it sends its MSG that occupies *z* access slots. Let *t* (1≤t≤z) denote a time index of the access slots used by delivered MSG. Additionally, let $n_{p,t}$ and $n_{m,t}$ denote the number of SNs transmitting a preamble and the number of SNs transmitting MSG at access slot *t*, respectively. In MT, the desired MSG from an SN is received by HS through *L* signal paths. A collision-outage is assumed to occur if the received power, from SN-A for example, of at least one path drops below the power from other SNs that use the same code. If the collision-outage occurs, the desired MSG is corrupted by other MSGs. Recalling that *w* denotes the number of SNs that receive the same AI with SN-A, the probability of safe reception of MSG from SN-A without collision-outage at HS is given by:

(7) $$\begin{array}{c} P_{A}^{\left(\mathrm{wco}\right)}=\prod_{l=1}^{L}\mathrm{Pr}\left\{P_{A}^{\left(m,l\right)}>\max_{i=1,2,\cdots ,w}P_{i}^{\left(m,l\right)}\right\}, \end{array}$$

where $P_{i}^{\left(m,l\right)}$ is the received MSG power at the *l*-th finger from SN-*i*. It is noted that $P_{i}^{\left(m\right)}=\sum_{l=1}^{L}P_{i}^{\left(m,l\right)}$ for all *i*. A closed-form expression of $P_{A}^{\left(\mathrm{wco}\right)}$ is presented in Appendix A.

Since a code jammer behaves similarly to legitimate SNs, it causes further interference additional to the interfering powers in the right-hand side of (7). With code jamming, the above collision-outage probability then becomes:

(8) $$\begin{array}{c} P_{A}^{\left(\mathrm{wco},c\right)}=\prod_{l=1}^{L}\mathrm{Pr}\left\{P_{A}^{\left(m,l\right)}>\max\left(\max_{i=1,2,\cdots ,w}\left(P_{i}^{\left(m,l\right)}\right),P_{J}^{\left(m,l\right)}\right)\right\}, \end{array}$$

where $P_{J}^{\left(m,l\right)}$ is the received power of the *l*-th finger from the code jammer that transmits a fake message. If the jammer cannot receive the AI, then it is assumed to transmit a fake preamble and hence works like a power jammer. A closed-form expression of $P_{A}^{\left(\mathrm{wco},c\right)}$ is presented in Appendix B.

Let $P_{i,t}^{\left(p\right)}$ and $P_{j,t}^{\left(m\right)}$ be the total received power from SN-*i* transmitting a preamble and SN-*j* transmitting MSG (using the different access codes from SN-A) at the *t*-th (1≤t≤z) access slot, respectively. Let $n_{p,t}$ and $n_{m,t}$ be the corresponding numbers of SNs transmitting a preamble and MSG at the *t*-th access slot, respectively. Then, the SINR of MSG signals from SN-A, $\gamma_{A}^{m}$, is defined by:

(9) $$\begin{array}{c} \gamma_{A}^{\left(m\right)}\overset{\left(\mathrm{def}\right)}{=}\frac{zP_{A}^{\left(m\right)}}{\sum_{t=1}^{z}\left(\sum_{i=1}^{n_{p,t}}P_{i,t}^{\left(p\right)}+\sum_{j=1}^{n_{m,t}}P_{j,t}^{\left(m\right)}\right)+z\left(\sum_{i=1}^{w}P_{i}^{\left(m\right)}+n_{J}P_{J}\right)+z\eta }. \end{array}$$

Thus, $\gamma_{A}^{\left(m\right)}\geq \beta_{m}$ is the condition for correct reception of MSG sent by SN-A, where $\beta_{m}$ is the SINR requirement. In this paper, since we model that condition $\gamma_{A}^{\left(m\right)}\geq \beta_{m}$ is applied only when collision-outage is free, $\gamma_{A}^{\left(m\right)}$ is equally obtained for power and code jamming.

### 2.4. Power Ramping for Preamble Transmission and Message Transmission

By using open-loop power estimation, an SN can adjust its initial transmission power based on the received signal strength from the HS [17]. The aim is to let the received power at the HS exceed a predetermined power level. For the initial PT, we assume that all SNs have the same target power level μL, recalling that μ is an average path power and *L* is the number of effective paths. If the initial PT fails, a higher target power level is used for retransmission. We assume that μL is the power increment unit adopted at each of preamble retransmissions as in [22]. Let us denote the number of retransmissions by *r*, and let $m_{r}\overset{\left(\mathrm{def}\right)}{=}r+1$. Then, $m_{r}\mu L$ represents the target power level in the *r*-th preamble retransmission. Hereafter, we denote the average number of preamble retransmissions by $\bar{m}$, and thus, $\bar{m}\mu L$ means that the average power level is used in PT. Average jamming power is also represented by $m_{J}$ (in the unit of power increment step μL), such that $m_{J}\mu L=P_{J}$, where $P_{J}$ is the maximum power level of jamming signals.

After PT is successful, SNs that received AI send MSG with an adjusted power level. Let us denote a power increasing factor for MSG transmission by α(≥1). When an SN receives AI successfully after *r* or the $\bar{m}$-th preamble retransmission, it sends its MSG using power level $\alpha m_{r}\mu L$ or $\alpha \bar{m}\mu L$, respectively.

## 3. RA Performance Analysis

### 3.1. Success Probability of Preamble Transmission

#### 3.1.1. Success Probability of *r*-th Preamble Retransmission

Given that $n_{p}$, $n_{m}$, *k* and $n_{J}$, let us denote the conditional success probability at the *r*-th preamble retransmission (i.e., at the (r+1)-th PT) by $u_{•}\left(r|n_{p},k,n_{m},n_{J}\right)$, where •∈{P,S} and furthermore *P* for power jamming and *S* for code jamming. The probability can be obtained analogously by the method used in [22] according to the criteria (4) and (5), respectively.

(10) $$\begin{array}{cc} & u_{•}\left(r|n_{p},k,n_{m},n_{J}\right)=\left\{\begin{array}{c} \frac{\exp\left(-\frac{\beta_{p}c}{\mu }\right)}{{\left(1+\beta_{p}\right)}^{b}\left(b-1\right)!}\sum_{i=0}^{a-1}\sum_{j=0}^{i}\frac{(b+j-1)!}{j!(i-j)!}\frac{\beta_{p}^{i}}{{\left(1+\beta_{p}\right)}^{j}}{\left(\frac{c}{\mu }\right)}^{i-j},1\leq k\leq n_{p}-1,\\ \exp\left(-\frac{\beta_{p}c}{\mu }\right)\sum_{i=0}^{a-1}\frac{1}{i!}{\left(\frac{\beta_{p}c}{\mu }\right)}^{i},\;k=n_{p}, \end{array}\right. \end{array}$$

where the three interim variables *a*, *b* and *c* for the power jamming (i.e., •=P) are defined as:

(11) $$\begin{array}{c} \left\{\begin{array}{c} a=\lfloor \left(k-1\right)\bar{m}L+m_{r}L\rfloor ,\\ b=\lfloor \left(n_{p}-k\right)\bar{m}L+n_{m}\alpha \bar{m}L+n_{J}m_{J}L\rfloor ,\\ c=\lfloor \eta \rfloor , \end{array}\right. \end{array}$$

and for the code jamming (i.e., •=C):

(12) $$\begin{array}{c} \left\{\begin{array}{c} a=\lfloor \left(k-1\right)\bar{m}L+m_{r}L+m_{J}L\rfloor ,\\ b=\lfloor \left(n_{p}-k\right)\bar{m}L+n_{m}\alpha \bar{m}L+\left(n_{J}-1\right)m_{J}L\rfloor ,\\ c=\lfloor \eta \rfloor . \end{array}\right. \end{array}$$

Aggregating $u_{•}\left(r|n_{p},k,n_{m},n_{J}\right)$ for all possible values on $n_{p}$ and *k* gives:

(13) $$\begin{array}{c} u_{•}\left(r|n_{m},n_{J}\right)=\sum_{n_{p}=1}^{\infty }\sum_{k=1}^{n_{p}}\mathrm{Pr}\left\{K=k|N=n_{p}\right\}\mathrm{Pr}\left\{N=n_{p}\right\}{\tilde{u}}_{•}\left(r|n_{p},k,n_{m},n_{J}\right), \end{array}$$

where an interim function ${\tilde{u}}_{•}\left(r|n_{p},k,n_{m},n_{J}\right)$ represents either ${\tilde{u}}_{P}\left(r|n_{p},k,n_{m},n_{J}\right)=u_{P}\left(r|n_{p},k,n_{m},n_{J}\right)$ for power jamming or:

(14) $$\begin{array}{cc} & {\tilde{u}}_{C}\left(r|n_{p},k,n_{m},n_{J}\right)=\underset{u_{C,1}}{\underset{︸}{\frac{n_{J}}{s}u_{C}\left(r|n_{p},k,n_{m},n_{J}\right)}}+\underset{u_{C,2}}{\underset{︸}{\frac{s-n_{J}}{s}u_{P}\left(r|n_{p},k,n_{m},n_{J}\right)}}, \end{array}$$

for code jamming. Furthermore, $u_{C,1}$ and $u_{C,2}$ in (14) represent whether SN-A selects the code being jammed by a code jammer or not, respectively. Hereafter, we use $u_{•}\left(r\right)\overset{\left(\mathrm{def}\right)}{=}u_{•}\left(r|n_{m},n_{J}\right)$ for notational brevity.

#### 3.1.2. Derivation of $\bar{m}$

Let $G_{0}$ and $G_{r}$ denote the arrival rates of the initial PT and the *r*-th preamble retransmissions, respectively. Let $S_{P}$ be the throughput of PT, and let $r_{\max}$ be the maximum number of preamble retransmissions allowed. Let $q_{A}$ denote the probability that SN-A receives AI successfully. The relationship between $G_{0}$ and $G_{r}$ is then:

(15) $$\begin{array}{c} \left\{\begin{array}{c} G_{0}=S_{P}+G_{r_{\max}}\left\{1-u_{•}\left(r_{\max}\right)q_{A}\right\},\\ G_{1}=G_{0}\left\{1-u_{•}\left(0\right)q_{A}\right\},\\ G_{2}=G_{1}\left\{1-u_{•}\left(1\right)q_{A}\right\},\\ \;\;\;\;\;\vdots \\ G_{r_{\max}}=G_{r_{\max}-1}\left\{1-u_{•}\left(r_{\max}-1\right)q_{A}\right\}. \end{array}\right. \end{array}$$

The composite offered traffic is therefore $G=\sum_{r=0}^{r_{\max}}G_{r}$. From (15), $G_{r}$ is given by:

(16) $$\begin{array}{c} G_{r}=\frac{G\prod_{i=0}^{r-1}\left\{1-u_{•}\left(i\right)q_{A}\right\}}{1+\sum_{j=1}^{r_{max}}\prod_{i=0}^{j-1}\left\{1-u_{•}\left(i\right)q_{A}\right\}}. \end{array}$$

The average number of retransmissions $\bar{m}$ to measure the corresponding average power level in the unit of μL is then given by:

(17) $$\bar{m}=\sum_{r=0}^{r_{\max}}m_{r}\frac{G_{r}}{G}=\frac{1+\sum_{r=1}^{r_{\max}}\left[m_{r}\prod_{i=0}^{r-1}\left\{1-u_{•}\left(i\right)q_{A}\right\}\right]}{1+\sum_{r=1}^{r_{\max}}\prod_{i=0}^{r-1}\left\{1-u_{•}\left(i\right)q_{A}\right\}}.$$

It should be noted the success probability $u_{•}\left(r\right)$ in (13) is a function of $\bar{m}$, while $\bar{m}$ as expressed in (17) is a function of $u_{•}\left(r\right)$. Therefore, (13) and (17) are to be solved recursively in order to get $\bar{m}$.

### 3.2. Throughput of Random Access Request

Let $q_{J}$ denote the probability that a jammer receives AI successfully. The success probability of MT after the successful *r*-th preamble retransmission in the presence of the power jamming and the code jamming for given $n_{m}$, $n_{m,t}$, $n_{p,t}$ and $n_{J}$ can be derived as:

(18) TP(r|nm,nm,t,np,t,nJ)=∑np=1∞∑k=1npPr{K=k|N=np}Pr{N=np}uP(r|np,k,nm,nJ)qA×∑w=0k−1k−1wqw(1−q)k−1−wPA(wco)︸(I)Pr{γA(m)≥βm|nm,t,np,t,w,nJ}︸(II),

and:

(19) TC(r|nm,nm,t,np,t,nJ)=∑np=1∞∑k=1npPr{K=k|N=np}Pr{N=np}qA∑w=0k−1k−1wqw(1−q)k−1−w×[nJsuC(r|np,k,nm,nJ)qJPA(wco,c)︸(III)+(1−qJ)PA(wco)+s−nJsuP(r|np,k,nm,nJ)PA(wco)]Pr{γA(m)≥βm|nm,t,np,t,w,nJ},

respectively. Hereafter, we use $T_{•}\left(r\right)\overset{\left(\mathrm{def}\right)}{=}T_{•}\left(r|n_{m},n_{m,t},n_{p,t},n_{J}\right)$ for notational brevity. Practically, the terms (I) (also (III)) and (II) are not independent when w≥1 and a closed-form expression of (18) (also for (19)) is not tractable. In this paper, we assume that the terms are independent for brevity. If w=0, (I) becomes one, and the independent assumption is valid. Now, the term (II) can be derived based on the result in (10):

(20) $$\begin{array}{c} \left(\mathrm{II}\right)=\frac{\exp\left(-\frac{\beta_{m}c}{\mu }\right)}{{\left(1+\beta_{m}\right)}^{b}\left(b-1\right)!}\sum_{i=0}^{a-1}\sum_{j=0}^{i}\frac{(b+j-1)!}{j!(i-j)!}\frac{\beta_{m}^{i}}{{\left(1+\beta_{m}\right)}^{j}}{\left(\frac{c}{\mu }\right)}^{i-j}, \end{array}$$

where the three interim variables *a*, *b* and *c* are defined as:

(21) $$\begin{array}{c} \left\{\begin{array}{c} a=\lfloor z\alpha m_{r}L\rfloor ,\\ b=\lfloor \sum_{t=1}^{z}\left(n_{p,t}\bar{m}L+n_{m,t}\alpha \bar{m}L\right)+z\left(w\alpha \bar{m}L+n_{J}m_{J}L\right)\rfloor ,\\ c=\lfloor z\eta \rfloor . \end{array}\right. \end{array}$$

From (18) and (19), the throughput of the preamble and the message transmission can be derived as:

(22) $$\begin{array}{ccc} S_{T} & = & \sum_{r=0}^{r_{max}}G_{r}T_{•}\left(r\right)=\sum_{r=0}^{r_{max}}\frac{G\prod_{i=0}^{r_{max}-1}\left\{1-u_{•}\left(i\right)q_{A}\right\}T_{•}\left(r\right)}{1+\sum_{j=1}^{r_{max}}\prod_{i=0}^{j-1}\left\{1-u_{•}\left(i\right)q_{A}\right\}}. \end{array}$$

### 3.3. Access Delay of Random Access Request

The success of TC consists of both successes of PT and MT. The failure of TC then occurs when the number of preamble retransmissions exceeds $r_{max}$ or MT fails after the SN receives AI. If TC is declared to be a failure, SNs restarts PT in a new TC with setting the transmitting power at an initial level, i.e., $m_{r}=1$. Let *C* be the number of restarts of TC to an end of successful MT. By assuming that all transmission attempts are independent, the distribution of *C* is given by:

(23) $$\begin{array}{c} \mathrm{Pr}\left\{C=c\right\}=\frac{S_{T}}{G}{\left(1-\frac{S_{T}}{G}\right)}^{c},\;\;\;\;c=0,1,2,\cdots . \end{array}$$

Let ${\bar{T}}_{•}^{\left(M\right)}\left(r|n_{m},n_{m,t},n_{p,t},n_{J}\right)$ be the probability that MT has failed though PT (i.e., the *r*-th retransmission), and receiving AI are successful, given $n_{m}$, $n_{m,t}$, $n_{p,t}$ and $n_{J}$. ${\bar{T}}_{•}^{\left(M\right)}\left(r\right)\overset{\left(\mathrm{def}\right)}{=}{\bar{T}}_{•}^{\left(M\right)}\left(r|n_{m},n_{m,t},n_{p,t},n_{J}\right)$ can be expressed as in (24) and (25) for power and code jamming, respectively.

(24) T¯P(r|nm,nm,t,np,t,nJ)=∑np=1∞∑k=1npPr{K=k|N=np}Pr{N=np}uP(r|np,k,nm,nJ)qA×∑w=0k−1k−1wqw(1−q)k−1−w1−PA(wco)Pr{γA(m)≥βm|nm,t,np,t,w,nJ},

(25) T¯C(r|nm,nm,t,np,t,nJ)=∑np=1∞∑k=1npPr{K=k|N=np}Pr{N=np}qA∑w=0k−1k−1wqw(1−q)k−1−w×[nJsuC(r|np,k,nm,nJ)qJ1−PA(wco,c)Pr{γA(m)≥βm|nm,t,np,t,w,nJ}+nJsuC(r|np,k,nm,nJ)(1−qJ)+s−nJsuP(r|np,k,nm,nJ)×1−PA(wco)Pr{γA(m)≥βm|nm,t,np,t,w,nJ}].

Let us denote the average delay (in the unit of access slot) occurring in PT and receiving AI by $D_{P}$. Let $D_{S}$ and $D_{F}$ be the average delay occurred in TC when TC terminates successfully or not, respectively. By using (19) and (25) for $D_{S}$, and (18) and (24) for $D_{F}$, we can have, in addition to (16),

(26) $$\begin{array}{ccc} D_{F} & = & \frac{1}{\Omega_{F}}\left[\sum_{r=0}^{r_{max}}\frac{G_{r}}{G}{\bar{T}}_{•}\left(r\right)\left\{\left(r+1\right)D_{P}+z\right\}+\left\{1-\sum_{r=0}^{r_{max}}\frac{G_{r}}{G}\left({\bar{T}}_{•}\left(r\right)+T_{•}\left(r\right)\right)\right\}\left(r_{max}+1\right)D_{P}\right], \end{array}$$

where $\Omega_{F}=1-\sum_{r=0}^{r_{max}}\frac{G_{r}}{G}T_{•}\left(r\right)$, and:

(27) $$\begin{array}{ccc} D_{S} & = & \frac{1}{\Omega_{S}}\sum_{r=0}^{r_{max}}\frac{G_{r}}{G}T_{•}\left(r\right)\left\{\left(r+1\right)D_{P}+z\right\}, \end{array}$$

where $\Omega_{S}=\sum_{r=0}^{r_{max}}G_{r}T_{•}\left(r\right)/G$.

By using (23), (26) and (27), the total access delay $D_{T}$ is given by:

(28) $$\begin{array}{c} D_{T}=\sum_{c=0}^{\infty }\mathrm{Pr}\left\{C=c\right\}\left(cD_{F}+D_{S}\right). \end{array}$$

## 4. Numerical Results

Computer simulation is done using the parameter values given in Table 1. The number of SNs transmitting preamble signals follows a Poisson distribution with mean *G*, and we assume that $n_{m}=n_{m,t}=n_{p,t}=G$ for all *t*. Thus, when G≥2, it is assumed in the simulation that SNs during PT are interfered by other multiple SNs that are transmitting either MSG or preamble. Additionally, scenarios with a different number of jammers $n_{J}=1,2,3$ are also tested, respectively.

**Figure 2:**
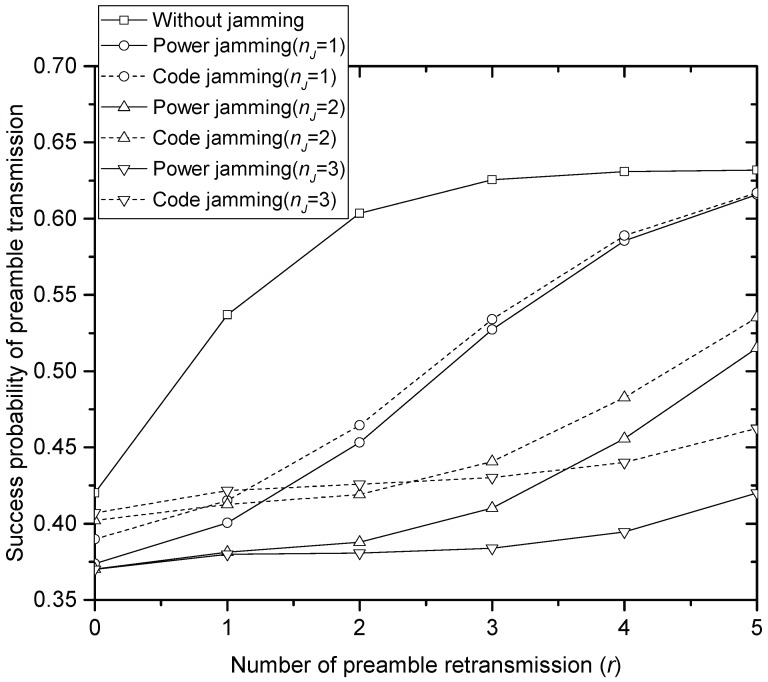
Success probability of the preamble transmission when *G* = 1.

**Figure 3:**
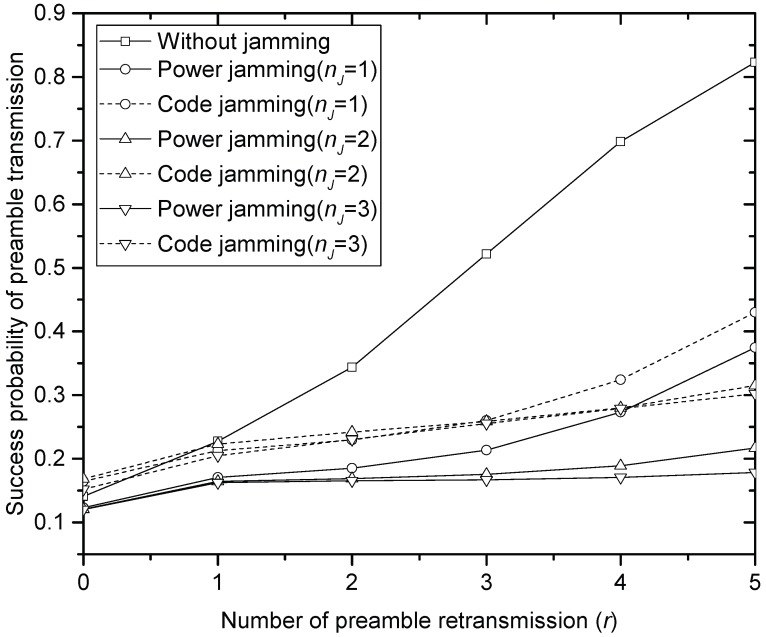
Success probability of the preamble transmission when *G* = 3.

**Figure 4:**
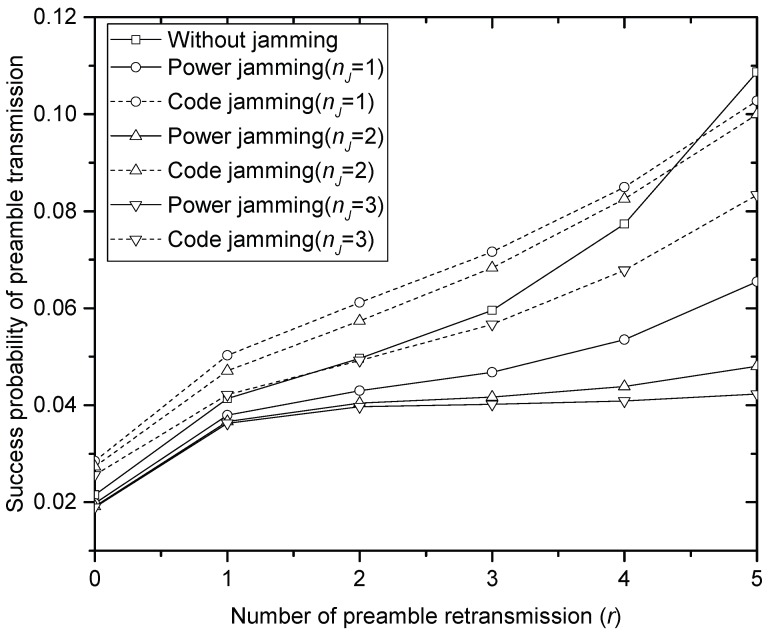
Success probability of the preamble transmission when *G* = 5.

Figure 2, Figure 3 and Figure 4 compare the success probability $u_{•}\left(r\right)$ of PT under different jamming attacks by the different numbers of jammers when the offered traffic *G* is 1, 3 and 5, respectively. In the figures, it is seen that the code jamming is less harmful than the power jamming in terms of the success probability. This is because, referring to (5), the signal power of the code jammer is added to desired signal power from SNs that select the same access code with the jammer, which increases $u_{•}\left(r\right)$ at HS. In power jamming, on the other hand, the whole power is treated as interference. In the same context, it is seen that the performance definitely degrades as the number of power jammers increases in Figure 2, Figure 3 and Figure 4. In code jamming, however, more jammers can contribute to improving the performance: for example, if the number of code jammers increases to 1, 2 and 3, in Figure 2 (when *G* = 1), $u_{•}\left(0\right)$ also increases to 0.38, 0.4 and 0.41, respectively. It should be noted that the code jammers do not always contribute to increasing $u_{•}\left(r\right)$: for example, when r≥3 in Figure 2, it is seen that the more code jammers provide the lower $u_{•}\left(r\right)$, which is also true for the overall range of tested *r* in Figure 4 (when *G* = 5). Furthermore, code jamming (with one or two jammers) sometimes (when 0≤r≤4) gives better performance than PT without jamming especially when *G* = 5 in Figure 4. Comparing Figure 2, Figure 3 and Figure 4, it is seen that jamming severely degrades the performance of $u_{•}\left(r\right)$ especially in the light offered load. In the very heavy load, code jamming sometimes increases $u_{•}\left(r\right)$. In terms of $u_{•}\left(r\right)$, power jamming is more harmful than code jamming over a wide range of parameter values.

**Figure 5:**
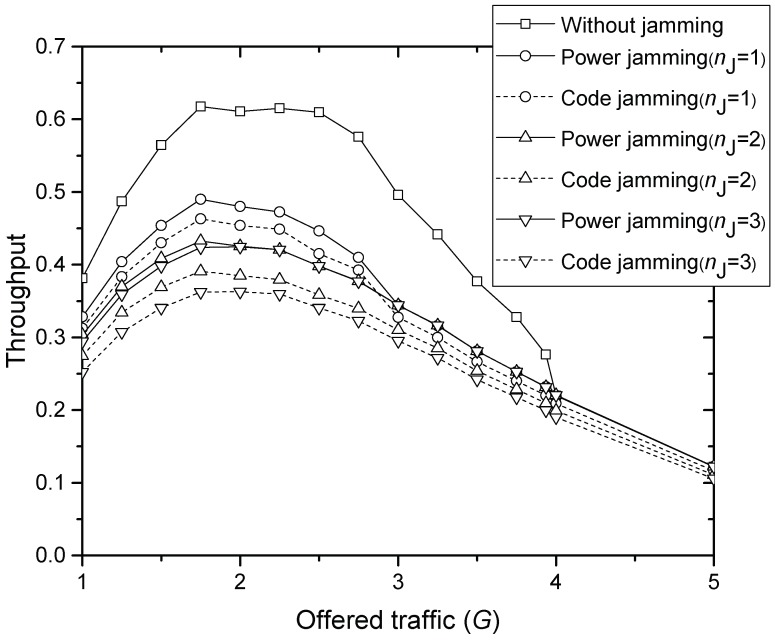
Throughput of the message transmission.

Figure 5 compares the throughput of final MSG transmission $S_{T}$ for increasing offered traffic *G*, where code and power jamming attacks with 1–3 jammers are considered, respectively. If the same number of jammers is considered, the throughput with code jamming is smaller than that with power jamming over all *G* tested, though code jamming achieves usually better $u_{•}\left(r\right)$ than power jamming in Figure 2, Figure 3 and Figure 4. This is because MSGs from desired SNs have collided with those from the code jammers using the same code. When G=2, 21%, 25%, 30%, 36%, 30% and 40% of throughput are lost due to 1 power jammer, 1 code jammer, 2 power jammers, 3 power jammers, 2 code jammers and 3 code jammers, respectively. When G=3, the amount of throughput loss is not distinguishable between the numbers of power jammers. Moreover, when G=4, the throughput with and without power jamming becomes equal due to the overwhelming interference. However, code jammers further degrade the throughput compared to power jammers over all *G* tested.

**Figure 6:**
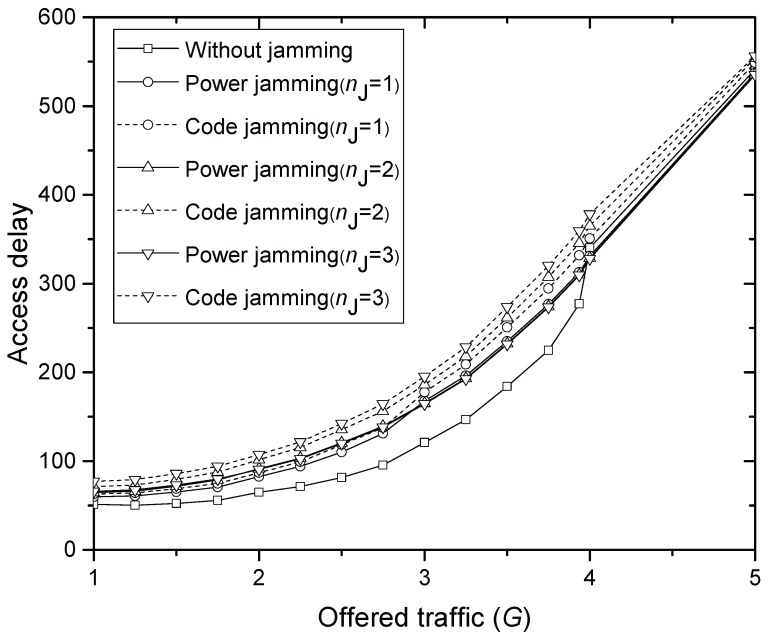
Average delay in the message transmission.

Figure 6 compares the access delay $D_{T}$ of final MSG transmission for increasing offered traffic *G*, where code and power jamming attacks with 1–3 jammers are considered, respectively. The result in Figure 6 certainly matches that in Figure 5. If the same number of jammers is considered, the delay with code jamming is greater than that with power jamming over all *G* tested, which is a similar trend that can be expected from Figure 5. When G=2, 27%, 34%, 40%, 56%, 40% and 65% of greater delay than without-jamming result due to 1 power jammer, 1 code jammer, 2 power jammers, 3 power jammers, 2 code jammers and 3 code jammers, respectively. When G=3, the amount of delay increase is not distinguishable with the numbers of power jammers. Moreover, when G=4, the delay with and without power jamming becomes equal due to overwhelming interference. However, code jammers further increase the delay compared to power jammers over all *G* tested.

From the above numerical investigations, we can address two ideas to detect whether the jammers exist or not if HS has certain measurement data, the implementation of which would be left for future study. One is using the concave throughput graphs achieved in Figure 5. We assume that HS have measurement data on received power (RxP) vs. throughput without jammers. Since the offered load cannot be directly measured, RxP is used to estimate the load. The jammer seems to generate additional interference, by which HS may overestimate the offered load since it has accordingly low throughput. For example, with one power jammer, the throughput in Figure 5 is about 0.47 when G=2.0, which can be achieved when either G=1.2 or 3.0 if the jammer does not exist. Let us assume that 0.47 is the current throughput seen at HS. If HS has the measurement data, then HS can judge whether current 0.47 is due to the low load or possibly due to the jammer. Let us denote the RxP measured when the throughput is 0.47 by $R_{1}$ and $R_{2}$ ($R_{1}\leq R_{2}$), which represent two different loads G=1.2 and 3.0 (noting that both provide the same throughput 0.47), respectively. Of course, HS does not know *G* exactly, but understands that throughput 0.47 is achieved by different load conditions: low and high, respectively. If current RxP is approximately equal to $R_{1}$, then the throughput is due to low load. However, if it is much greater than $R_{1}$, HS sends a signaling message that temporarily prevents each SN from sending preambles with certain probability p(0<p<1). Then, the load should be increased if throughput 0.47 is due to the high load. Otherwise, the load should be decreased, and there probably exits a jammer.

The other idea is using the monotonically increasing delay graphs in Figure 6. If HS is assumed to have measurement data on RxP vs. delay without jammers, then it can guess that the current excessive delay is possibly due to jamming powers. Other methods to avoid or detect jamming such as using directional antennas are intensively summarized in [23].

## 5. Conclusions

We have investigated RA performances in a WSN where power or code jammers actively disturb the information transmission from distributed SNs to a central HS. Average throughput and average delay for the information message are modeled, respectively. Numerical investigation shows that power jamming is more harmful than code jamming in the stage of PT. However, code jamming finally degrades the modeled performances more severely. Code jamming falsely attracts HS to send AI and thus further deteriorates the resource allocation in the WSN. As a remedy of code jamming, a new secure code, which is different from the access code used in PT and hardly known to code jammers, can be used in sending MSG. This requires a more sophisticated protocol to allocate the secure codes, which is left for future work. The model and results in this paper are certainly useful in building up better and more sophisticated RA methods that tolerate attacks from malicious transmitters.

## Figures and Tables

**Table 1:** Parameter values used in numerical evaluation.

Parameter	Value
The number of resolvable paths	L=3
Average received power from one path	μ=1
Interference plus noise power	η=2
Minimum SINR for correct preamble reception	$\beta_{p}=-5$ dB
Power increment ratio for message transmission	α=1.2
Minimum SINR for correct message reception	$\beta_{m}=-5$ dB
Maximum number of preamble retransmission	$r_{max}=5$
The number of available access codes	s=16
Probability of successfully receiving AI by an SN or a jammer	$q=q_{A}=q_{J}=0.8$
Average delay in reception of AI after successful PT	$D_{P}=3$
Slot length in message transmission	z=15

## Abbreviations

The following abbreviations are used in this manuscript:

AI	Indicator of acquisition
AICH	AI channel
AN	Artificial noise
DoS	Denial of service
HS	Head sensor
LTE	Long-Term Evolution
MSG	Frame-long message
MT	MSG transmission
PRACH	Physical RACH
PT	Preamble transmission
RA	Random access
RACH	RA channel
RF	Radio frequency
SINR	Signal-to-interference-plus-noise ratio
SN	Sensor node
SN-A	Anonymous SN
TC	Transmission cycle
UMTS	Universal Mobile Telecommunication System
WCDMA	Wideband Code Division Multiple Access
WSN	Wireless sensor network

## Appendix A Derivation of $P_{A}^{\left(\mathrm{wco}\right)}$

Let $P_{i}^{\left(m,l\right)}∼\exp\left(\alpha \bar{m}\mu \right)$, i=1,2,…,w, be statistically independent and identically distributed (i.i.d.) exponential r.v.’s with mean $\alpha \bar{m}\mu$. Additionally, $P_{A}^{\left(m,l\right)}∼\exp\left(\alpha m_{r}\mu \right)$ follows the exponential distribution with mean $\alpha m_{r}\mu$. Therefore, the i.i.d finger path is considered as:

(A1) PrPA(m,l)>maxi=1,2,⋯,wPi(m,l)=∫0∞1−e−tαm¯μw1αmrμe−tαmrμdt=∑v=0wwv−1v1αmrμ∫0∞e−vαm¯μ+1αmrμtdt=∑v=0wwv−1v1αmrμ·1vαm¯μ+1αmrμ.

According to order statistics [24],

(A2) PA(wco)=∏l=1LPrPA(m,l)>maxi=1,2,⋯,wPi(m,l)=∑v=0wwv−1vm¯vmr+m¯L.

## Appendix B Derivation of $P_{A}^{\left(\mathrm{wco},c\right)}$

Let $P_{J}^{\left(m,l\right)}∼\exp\left(m_{J}\mu \right)$ be also an exponential distribution with mean $m_{J}\mu$. Using the same approach as above,

(A3) PrPA(m,l)>maxi=1,2,⋯,wPi(m,l),PJ(m,l)=∫0∞1−e−tαm¯μw1−e−tmJμ1αmrμe−tαmrμdt=∑v=0wwv−1v1αmrμ∫0∞e−vαm¯μ+1αmrμt−e−vαm¯μ+1αmrμ+1mJμtdt=∑v=0wwv−1v1αmrμ1vαm¯μ+1αmrμ−1vαm¯μ+1αmrμ+1mJμ.

According to order statistics [24],

(A4) PA(wco,c)=∏l=1LPrPA(m,l)>maxi=1,2,⋯,wPi(m,l),PJ(m,l)=∑v=0wwv−1vm¯vmr+m¯−m¯vmr+m¯+αmrm¯mJL.

## Appendix C Notations

**Table A1:** Notations.

Notation	Description
*N*	The number of legitimate SNs contending for the same access slot
*G*	Arrival rate of composite PT is modeled by a Poisson process
*s*	The number of available access codes
*K*	The number of SNs selecting the same code in the same time slot
*L*	The number of fingers in a RAKEreceiver
μ	Average received power from each path
$P^{\left(l\right)}$	Received power at each finger
$n_{p}$	The number of SNs in simultaneous PT
$n_{J}$	The number of jammers
$n_{m}$	The number of SNs transmitting MSG
$P_{i}^{\left(p\right)}$	Received power from SN-*i* transmitting a preamble
$P_{j}^{\left(m\right)}$	Received power from SN-*j* transmitting MSG
$P_{J}$	Received power from a hostile jammer
$\beta_{p}$	Minimum SINR required for successfully decoding a preamble
η	Interference plus noise power
*q*	Probability of successful reception of AI
*W*	The number of SNs that successfully receive AI
*z*	Slot length for MSG transmission
*t*	Time index of the access slots used by delivering MSG
$n_{p,t}$	The number of SNs transmitting a preamble at access slot *t*
$n_{m,t}$	The number of SNs transmitting MSG at access slot *t*
*w*	The number of SNs that receive the same AI with SN-A
$P_{A}^{\left(wco\right)}$	Probability of safe reception of MSG without collision-outage
$P_{i}^{\left(m,l\right)}$	Received MSG power at *l*-th finger from SN-*i*
$P_{i,t}^{\left(p\right)}$	Received power from SN-*i* transmitting a preamble at the *t*-th access slot
$P_{j,t}^{\left(m\right)}$	Received power from SN-*j* transmitting MSG at the *t*-th access slot
$\gamma_{A}^{\left(m\right)}$	SINR of MSG signals
$\beta_{m}$	SINR requirement of MSG signals
μ	Average path power
*r*	The number of retransmissions
$\bar{m}$	Average number of preamble retransmissions
$m_{J}$	Average jamming power
$P_{J}$	Maximum power level of jamming signals
α	Power increment ratio for MSG transmission
$u_{•}\left(r|n_{p},k,n_{m},n_{J}\right)$	Conditional success probability at the *r*-th preamble transmission
*P*	State variable for indicating power jamming
*S*	State variable for indicating code jamming
$G_{0}$	Arrival rate of initial PT
$G_{r}$	Arrival rate of the *r*-th preamble retransmissions
$S_{P}$	Throughput of PT
$r_{max}$	The maximum number of allowable preamble retransmissions
$q_{A}$	Probability that SN-A receives AI successfully
$q_{J}$	Probability that a jammer receives AI successfully
$T_{•}\left(r\right)$	Success probability of MT after the successful r-th preamble retransmission
$S_{T}$	Throughput of PT and MSG transmission
*C*	The number of restarts of TC
${\bar{T}}_{•}\left(r\right)$	Probability that MT is failed
$D_{P}$	Average delay occurred in PT and receiving AI
$D_{S}$	Average delay occurred in TC when TC terminates successfully
$D_{F}$	Average delay occurred in TC when TC terminates with a failure
$D_{T}$	Total access delay
$\overset{\left(\mathrm{def}\right)}{=}$	Definition declaration
⌊·⌋	Floor function

## References

[1] Akyildiz I.F., Su W., Sankarasubramaniam Y., Cayirci E. (2002). Wireless Sensor Networks: A Survey. Comput. Netw..

[2] Xu W., Ma K., Trappe W., Zhang Y. (2006). Jamming Sensor Networks: Attack and Defense Strategies. IEEE Netw..

[3] Shi E., Perrig A. (2004). Designing Secure Sensor Networks. Wirel. Commun. Mag..

[4] Wood A.D., Stankovic J.A. (2002). Denial of Service in Sensor Networks. Computer.

[5] Xu W., Trappe W., Zhang Y., Wood T. The Feasibility of Launching and Detecting Jamming Attacks in Wireless Networks. Proceedings of the 6th ACM International Symposium on Mobile Ad Hoc Networking and Computing.

[6] Li M., Koutsopoulos I., Poovendran R. (2010). Optimal Jamming Attack Strategies and Network Defense Policies in Wireless Sensor Netwroks. IEEE Trans. Mob. Comput..

[7] Aziz F.M., Shamma J.S., Stüber G.L. Resilience of LTE Networks Against Smart Jamming Attacks. Proceedings of the Global Communications Conference (GLOBECOM).

[8] Lichtman M., Jover R.P., Labib M., Rao R., Marojevic V., Reed J.H. (2016). LTE/LTE-A Jamming, Spoofing, and Sniffing: Threat Assessment and Mitigation. IEEE Commun. Mag..

[9] Del-Valle-Soto C., Mex-perera C., Monroy R., Nulazo-flores J.A. (2015). On Routing Protocol Influence on the Resilience of Wireless Sensor Networks to jamming attacks. Sensors.

[10] Li X., Dai H.-N., Wang H., Xiao H. (2016). On Performance Snalysis of Protective Jamming Schemes in Wireless Sensor Networks. Sensors.

[11] Han Z., Marina N., Debbah M., Hjørungnes A. (2009). Physical Layer Security Game: Interaction Between Source, Eavesdropper, and Friendly Jammer. EURASIP J. Wirel. Commun. Netw..

[12] Vilela J.P., Bloch M., Barros J., McLaughlin S.W. (2011). Wireless Secrecy Regions with Friendly Jamming. IEEE Trans. Inf. Forensics Secur..

[13] Yang M., Zhang B., Huang Y., Yang N., Guo D., Gao B. (2016). Secure Multiuser Communications in Wireless Sensor Networks with TAS and Cooperative Jamming. Sensors.

[14] Li Z., Jing T., Ma L., Huo Y., Qian J. (2016). Worst-Case Cooperative Jamming for Secure Communications in CIoT Networks. Sensors.

[15] ZigBee Alliance. http://www.zigbee.org.

[16] Gutierrez J.A., Callaway E.H., Barrett R. (2003). IEEE 802.15.4 Low-Rate Wireless Personal Area Networks.

[17] 3GPP TS 25.211 (V6.10.0) Physical Channels and Mapping of Transport Channels onto Physical Channels (FDD). Technical Specification (Release 6), Technical Specification Group Radio Access Network. 3GPP, 2003. http://www.qtc.jp/3GPP/Specs/25211-6a0.pdf.

[18] Labib M., Marojevic V., Reed J. Analyzing and Enhancing the Resilience of LTE/LTE-A Systems to RF Spoofing. Proceedings of the IEEE Conference Standards for Communications and Networking.

[19] 3GPP TS 25.214 (V6.5.0) Physical Layer Procedures (FDD). Technical Specification (Release 6), Technical Specification Group Radio Access Network. 3GPP, 2003. http://www.etsi.org/deliver/etsi_ts/125200_125299/125214/06.05.00_60/ts_125214v060500p.pdf.

[20] Moberg J., Löfgren M., Karlsson R.S. Throughput of The WCDMA Random Access Channel. Proceedings of the IST Mobile Communication Summit.

[21] Davis D.H., Gronemeyer S.A. (1980). Performance of Slotted ALOHA Random Access with Delay Capture and Randomized Time of Arrival. IEEE Trans. Commun..

[22] Yang Y., Yum T.-S.P. (2005). Analysis of Power Ramping Schemes for UTRA-FDD Random Access Channel. IEEE Trans. Wirel. Commun..

[23] Curiac D.-I. (2016). Wireless Sensor Network Security Enhancement Using Directional Antennas: State of the Art and Research Challenges. Sensors.

[24] David H.A. (1981). Order Statistics.

